# Initial Gastrectomy Indication Is Associated with Divergent Temporal Patterns of Remnant Gastric Cancer After Billroth II Reconstruction: A Retrospective Study

**DOI:** 10.3390/jcm15207933

**Published:** 2026-10-14

**Authors:** Yining Sun, Bingcan Yang, Feifan Chen, Kai Deng, Jinlin Yang, Zhu Wang

**Affiliations:** 1 Department of Gastroenterology and Hepatology, West China Hospital, Sichuan University, 37# Guoxue Lane, Chengdu 610041, China; yining.sun@wchscu.edu.cn (Y.S.); chenfeifan1993@wchscu.cn (F.C.); dengkai@wchscu.cn (K.D.); yangjinlin@wchscu.cn (J.Y.); 2 Department of Gastroenterology and Hepatology, West China Hospital, West China Xiamen Hospital, Sichuan University, Xiamen 361021, China; 15705950616@163.com

**Keywords:** remnant gastric cancer, Billroth II reconstruction, gastrectomy, endoscopic surveillance, temporal heterogeneity

## Abstract

**Background:** Remnant gastric cancer (RGC) may develop over markedly different intervals after gastrectomy. We investigated the time-dependent clinicopathological patterns of RGC after Billroth II reconstruction and their implications for endoscopic surveillance. **Methods:** We retrospectively reviewed patients diagnosed with RGC at West China Hospital between October 2012 and October 2022. Patients were classified by the indication for initial gastrectomy (malignant or benign disease) and the interval to RGC detection (≤3 years, >3 to ≤10 years, and >10 years). **Results:** Sixty-one patients were included; 39 (63.9%) had tumors at the anastomotic site. Histology was classifiable in 48 patients, of whom 45 had undifferentiated carcinoma (93.8%; 73.8% of the total cohort). The interval to RGC detection was shorter in the carcinoma group than in the benign-disease group (4.8 ± 5.7 vs. 31.6 ± 12.9 years, *p* < 0.001). Eighteen of 36 patients (50.0%) in the carcinoma group were diagnosed within 3 years, whereas 23 of 25 patients (92.0%) in the benign-disease group were diagnosed after 10 years. All 18 RGCs diagnosed ≤3 years after the initial gastrectomy occurred in patients whose initial indication was gastric carcinoma. In exploratory unadjusted pairwise comparisons among patients with known distant-metastasis status, distant metastasis was more frequent in the ≤3-year group than in the >10-year group (11/17 [64.7%] vs. 6/25 [24.0%], *p* = 0.012), whereas surgery for RGC was less frequent (5.6% vs. 53.8%, *p* = 0.001). **Conclusions:** RGC after Billroth II reconstruction showed distinct temporal patterns. Associations with metastatic burden and surgical treatment were exploratory, and the effects of postoperative interval and initial indication could not be reliably separated. These hypothesis-generating findings require prospective validation before specific surveillance intervals can be recommended.

## 1. Background

Gastric cancer remains a major global health burden, with the highest incidence rates occurring in Eastern Asia [1]. Remnant gastric cancer (RGC) refers to gastric carcinoma detected in the remaining stomach after partial gastrectomy for benign or malignant disease. Although relatively uncommon, accounting for approximately 1–8% of gastric cancers, RGC is becoming increasingly relevant as postoperative survival improves and more patients live with a gastric remnant [2,3,4]. RGC is frequently detected at an advanced stage, and clinical outcomes are determined largely by tumor stage and the feasibility of curative treatment rather than by its remnant origin alone [4,5,6]. Therefore, appropriate endoscopic surveillance is important for improving early detection and treatment opportunities.

RGC is clinically heterogeneous, and its presentation may vary according to the indication for the initial gastrectomy, reconstruction method, and interval after surgery. Billroth II reconstruction results in prolonged exposure of the anastomosis and remnant gastric mucosa to duodenogastric reflux and has been associated with RGC at the anastomotic site, particularly after long postoperative intervals [2,3,7,8,9,10,11]. Previous studies have also shown that RGC is generally detected earlier after gastrectomy for gastric malignancy than after surgery for benign disease. However, most previous studies included mixed reconstruction methods or only patients who underwent subsequent surgery, potentially underrepresenting patients with advanced or unresectable disease.

Gastric cancers detected within the first few years after surgery for malignancy represent a particular clinical challenge because they may include previously unrecognized synchronous lesions, residual disease, local recurrence, or true metachronous primary cancers. The clinical profile and management implications of this early postgastrectomy subgroup remain insufficiently characterized, particularly among patients with Billroth II reconstruction. Therefore, this study investigated the time-dependent clinicopathological patterns of RGC after Billroth II reconstruction, with particular attention to cancers detected within 3 years after the initial surgery, and explored their potential implications for the design of future postgastrectomy surveillance studies.

## 2. Patients and Methods

### 2.1. Study Design and Participants

This single-center retrospective observational case series was conducted at West China Hospital, Sichuan University. Medical and endoscopic records were reviewed to identify patients diagnosed with remnant gastric cancer (RGC) between October 2012 and October 2022. Patients were eligible if they had previously undergone distal gastrectomy with Billroth II reconstruction for a benign or malignant gastric disease and subsequently had histologically confirmed carcinoma in the remnant stomach. RGC was operationally defined as gastric carcinoma detected in the remnant stomach after partial gastrectomy, irrespective of the indication for the initial surgery. The initial record search identified 369 candidate cases for RGC after Billroth II reconstruction. This was a case-based retrospective search rather than systematic screening of the entire postgastrectomy population. Patients were excluded for incomplete clinical follow-up records, an unknown indication for the initial gastrectomy, concomitant malignancies in other organ systems, positive histopathological margins at the initial gastrectomy, noncurative initial resection with macroscopic residual disease, or anastomotic carcinoma diagnosed within 1 year after the initial gastrectomy. Additional exclusions were neuroendocrine carcinoma, gastrointestinal stromal tumor, primary cholangiocarcinoma invading the anastomosis, lymphoma, an indeterminate tumor origin, or an uncertain interval between the initial gastrectomy and RGC diagnosis. All eligible cases identified during the study period were consecutively included. For cancers detected within 3 years after the initial gastrectomy for gastric carcinoma, available initial and subsequent pathological records and postoperative endoscopic records were reviewed. Final histopathological margins at the initial gastrectomy were verified as negative, and no macroscopic residual tumor was documented. Postoperative endoscopic examinations showing no detected tumor before the subsequent RGC diagnosis were also verified. Initial and subsequent tumors were compared using the recorded histological type and differentiation; this was a comparison of pathological records rather than a centralized review of paired histological slides. These findings provided supporting evidence but did not definitively establish an independent new primary origin or exclude local recurrence or previously missed synchronous lesions. The ≤3-year subgroup was therefore defined by the interval to detection rather than by confirmed metachronous origin. The study protocol was approved by the Biomedical Research Ethics Committee of West China Hospital, Sichuan University (Approval No. 2022-2002).

### 2.2. Data Collection and Study Groups

Demographic and clinical variables included sex, age at RGC diagnosis, indication for the initial gastrectomy, and the interval between the initial gastrectomy and RGC diagnosis. Endoscopic variables included tumor location, the extent of remnant gastritis, and the presence of bile reflux. Histopathological characteristics, tumor extent, distant metastasis, and treatment after RGC diagnosis, including whether surgery for RGC was performed, were obtained from medical, imaging, endoscopic, and pathological records. Surgery for RGC referred to surgical treatment after RGC diagnosis and excluded endoscopic resection. Surgical intent was categorized as curative, palliative, or unknown according to the available clinical and operative records. Reasons for not undergoing surgery were recorded separately. For patients whose initial gastrectomy was performed for gastric carcinoma, initial tumor characteristics were extracted from available pathological reports and clinical records. Final histopathological margin status was verified against the postoperative pathology reports from the initial gastrectomy. Initial staging information was incomplete because some operations had been performed at other hospitals or many years previously, historical reports could not be retrieved, only brief discharge summaries were available, or the original reports did not document all staging components. Information that could not be verified was recorded as unknown. Historical information on *H. pylori* testing and eradication, and complications following the initial gastrectomy, could not be systematically reconstructed from the available records.

Postoperative follow-up differed according to the indication for the initial gastrectomy. In routine clinical practice at our center, patients treated for gastric carcinoma were generally advised to undergo follow-up assessments every 6–12 months during the first 3 years and every 1–2 years thereafter, with endoscopy and/or imaging performed as clinically indicated. These intervals describe the general follow-up approach rather than a uniform endoscopic surveillance protocol followed by all patients. Patients who underwent gastrectomy for benign disease had no standardized routine endoscopic surveillance schedule, and examinations were generally prompted by symptoms or other clinical indications. Because some initial operations and follow-up examinations occurred at other hospitals and historical records were incomplete, actual endoscopic examination dates, frequency, and adherence could not be comprehensively reconstructed for the entire cohort.

The interval to RGC detection was calculated from the date of the initial gastrectomy to the date of histological diagnosis of RGC. Continuous follow-up duration after the initial gastrectomy could not be reliably determined for the entire cohort. Patients were categorized according to the indication for the initial gastrectomy as the carcinoma group or the benign-disease group. For the time-dependent analyses, patients were divided into three postoperative interval groups: ≤3 years, >3 to ≤10 years, and >10 years. These postoperative interval categories were used for descriptive temporal comparisons and were not intended to estimate time-specific RGC incidence or to define or recommend optimal surveillance intervals.

### 2.3. Endoscopic and Clinicopathological Definitions

Tumor location was classified as the anastomotic or non-anastomotic site according to the endoscopic report. Endoscopic gastritis was identified by conspicuous mucosal redness, edema, or erosion. The extent of gastritis was graded according to the classification proposed by Kubo et al. [12]: grade 0, no gastritis; grade 1, gastritis limited to the anastomosis; grade 2, an intermediate extent between grades 1 and 3; and grade 3, gastritis involving the entire remnant stomach. Extensive gastritis was defined as an extent grade of 2 or 3. Bile reflux was categorized as present or absent according to the endoscopic report; visible yellowish fluid in the remnant stomach was considered evidence of bile reflux.

Histological diagnosis was based on the surgical specimen when available and otherwise on endoscopic biopsy specimens. Histological diagnoses were recorded using WHO terminology [13] and were subsequently grouped for analysis into differentiated, undifferentiated, or unclassified categories according to the study-defined classification. The differentiated group included papillary adenocarcinoma and well- or moderately differentiated adenocarcinoma, whereas the undifferentiated group included poorly differentiated adenocarcinoma, undifferentiated adenocarcinoma, signet-ring cell carcinoma, and mucinous adenocarcinoma. Tumors were categorized as unclassified when the available pathological records were insufficient to determine the differentiation category.

Tumor extent was recorded separately as the T category, the presence or absence of lymph-node metastasis, and the presence or absence of distant metastasis, according to the seventh edition of the AJCC TNM staging system [14]. The seventh edition was selected as a common reference framework to maintain consistency in the assessment of tumor extent across this historical cohort, which included RGC diagnoses between 2012 and 2022. For patients who underwent surgical resection, pathological assessment was used. For patients who did not undergo surgery, clinical assessment was based on pretreatment contrast-enhanced imaging. Unavailable information was categorized as unknown rather than negative.

### 2.4. Statistical Analysis

Statistical analyses were performed using IBM SPSS Statistics, version 23.0 (IBM Corp., Armonk, NY, USA). Categorical variables are presented as numbers and percentages, and continuous variables are presented as the mean ± standard deviation. Comparisons of categorical variables were performed using Pearson’s χ^2^ test or exact tests, as appropriate; Fisher’s exact test or an appropriate exact extension was used when cell counts were sparse. Continuous variables were compared using the independent-samples Student’s t test for two groups and one-way analysis of variance for three groups. Unknown staging values were retained as “unknown” for descriptive reporting but were excluded from inferential analyses involving the corresponding staging variable; analyses were therefore based on available cases. Overall comparisons across the three postoperative interval groups were performed before exploratory pairwise comparisons. Pairwise comparisons between the ≤3-year and >10-year groups were not adjusted for multiple testing and should therefore be interpreted as exploratory. Effect estimates for key comparisons are presented with 95% confidence intervals (CIs).

Because of the modest sample size and sparse events, multivariable analyses were deliberately restricted to parsimonious exploratory models. Firth penalized logistic regression was performed using the STATS FIRTHLOG extension in IBM SPSS Statistics version 23.0 to examine the associations of postoperative interval with distant metastasis and surgery for RGC, adjusting for age and sex. The >10-year group was used as the reference category. Patients with unknown distant-metastasis status were excluded from the corresponding model. The indication for initial gastrectomy was not entered simultaneously with postoperative interval because of their strong association across the interval groups. Adjusted odds ratios (ORs) with 95% CIs are reported. All tests were two-sided, and *p* < 0.05 was considered statistically significant.

## 3. Results

### 3.1. Overall Clinicopathological Characteristics of RGC

Of the 369 candidate cases identified, 276 were excluded according to the clinical eligibility criteria described in the Methods, leaving 93 cases. The aggregate number excluded was available, but counts for the individual exclusion reasons could not be retrospectively reconstructed. A further 13 cases were excluded: two with neuroendocrine carcinoma, one with a gastrointestinal stromal tumor, one with primary cholangiocarcinoma invading the anastomosis, two with lymphoma, and seven with an indeterminate tumor origin. Of the remaining 80 cases, 19 were excluded because the interval between the initial gastrectomy and RGC diagnosis was uncertain. The final analysis included 61 consecutively identified eligible patients. The mean age was 62.0 ± 11.0 years; 44 patients (72.1%) were male. Grade 2 or grade 3 gastritis was observed in 51 patients (83.6%), and bile reflux was detected in 44 (72.1%). RGC was located at the anastomotic site in 39 patients (63.9%). Histological classification was available for 48 patients, of whom 45 (93.8%; 73.8% of the entire cohort) had undifferentiated carcinoma. Thirty-five patients (57.4%) were diagnosed with RGC within 10 years after the initial gastrectomy, including 18 (29.5%) diagnosed within 3 years. The indication for the initial gastrectomy was gastric carcinoma in 36 patients (59.0%) and benign disease in 25 (41.0%). Overall, 19 patients (31.1%) underwent surgery for RGC (Table 1). Among these 19 patients, 16 underwent surgery with curative intent, one underwent palliative surgery, and surgical intent could not be determined in two. Among the 42 patients who did not undergo surgery, the reasons or treatment circumstances were disease extent precluding resection in 22, an overall clinical assessment that the anticipated surgical benefits were outweighed by the risks in 11, and poor functional status in one. The reason for not undergoing surgery was undocumented in eight patients. Tumor location, T-category distribution, and lymph-node metastasis did not differ significantly between patients who underwent surgery for RGC and those who did not. Among the 57 patients with known distant-metastasis status, distant metastasis was more frequent among patients who did not undergo surgery than among those who did (18/38 [47.4%] vs. 3/19 [15.8%], *p* = 0.023). Among the 45 patients with undifferentiated RGC, 31 (68.9%) had documented T4 disease, 30 (66.7%) had lymph-node metastasis, 15 (33.3%) had distant metastasis, and 16 (35.6%) underwent surgery for RGC.

### 3.2. RGC Patterns According to the Indication for Initial Gastrectomy

According to the indication for the initial gastrectomy, the patients were classified into the carcinoma group (*n* = 36) and the benign-disease group (*n* = 25). The interval between the initial gastrectomy and RGC diagnosis was significantly shorter in the carcinoma group than in the benign-disease group (4.8 ± 5.7 vs. 31.6 ± 12.9 years; mean difference, −26.8 years; 95% CI, −31.7 to −21.9; *p* < 0.001). In the carcinoma group, 18 patients (50.0%) were diagnosed with RGC within 3 years, 15 (41.7%) between >3 and ≤10 years, and 3 (8.3%) after >10 years. In contrast, none of the patients in the benign-disease group were diagnosed within 3 years, whereas 2 (8.0%) were diagnosed between >3 and ≤10 years and 23 (92.0%) after >10 years. The interval distribution differed significantly between the two groups (*p* < 0.001). Patients in the carcinoma group were younger than those in the benign-disease group (59.0 ± 10.9 vs. 66.3 ± 9.8 years; mean difference, −7.3 years; 95% CI, −12.8 to −1.8; *p* = 0.01) and were less likely to undergo surgery for RGC (7/36, 19.4% vs. 12/25, 48.0%; OR, 0.27; 95% CI, 0.07–0.94; *p* = 0.03). No significant differences were observed between the groups in sex, extent of gastritis, bile reflux, tumor location, histological type, T-category distribution, lymph-node metastasis, or distant metastasis (all *p* > 0.05; Table 2). Among the 36 patients whose initial gastrectomy was performed for gastric carcinoma, histological classification of the initial tumor was available in 24 patients, including 23 with undifferentiated carcinoma and 1 with differentiated carcinoma. Information on the T category was available in 17 patients, of whom 13 had T4 disease and 4 had T1–T3 disease. Lymph-node status was available in 17 patients, including 14 with and 3 without lymph-node metastasis, while distant-metastasis status was available in 17 patients, including 3 with and 14 without distant metastasis. Final histopathological assessment confirmed negative resection margins in all 36 patients, consistent with the intraoperative frozen-section findings (Table 3).

### 3.3. RGC Characteristics According to the Interval from Initial Gastrectomy

Patients were classified into three groups according to the interval between the initial gastrectomy and RGC diagnosis: ≤3 years (*n* = 18), >3 to ≤10 years (*n* = 17), and >10 years (*n* = 26). Review of the short-interval cases confirmed negative final histopathological margins and no documented macroscopic residual tumor at the initial gastrectomy, together with postoperative endoscopic examinations showing no detected tumor before RGC diagnosis. Comparison of available pathological records showed similar histological descriptions in several patients and differences in the recorded subtype or differentiation in others. However, some initial reports lacked specific histological details, and complete paired pathological documentation was not available for all patients. Consequently, the available evidence did not permit definitive classification of every short-interval lesion as a new primary cancer, local recurrence, or a previously missed synchronous lesion.

**Figure 1:**
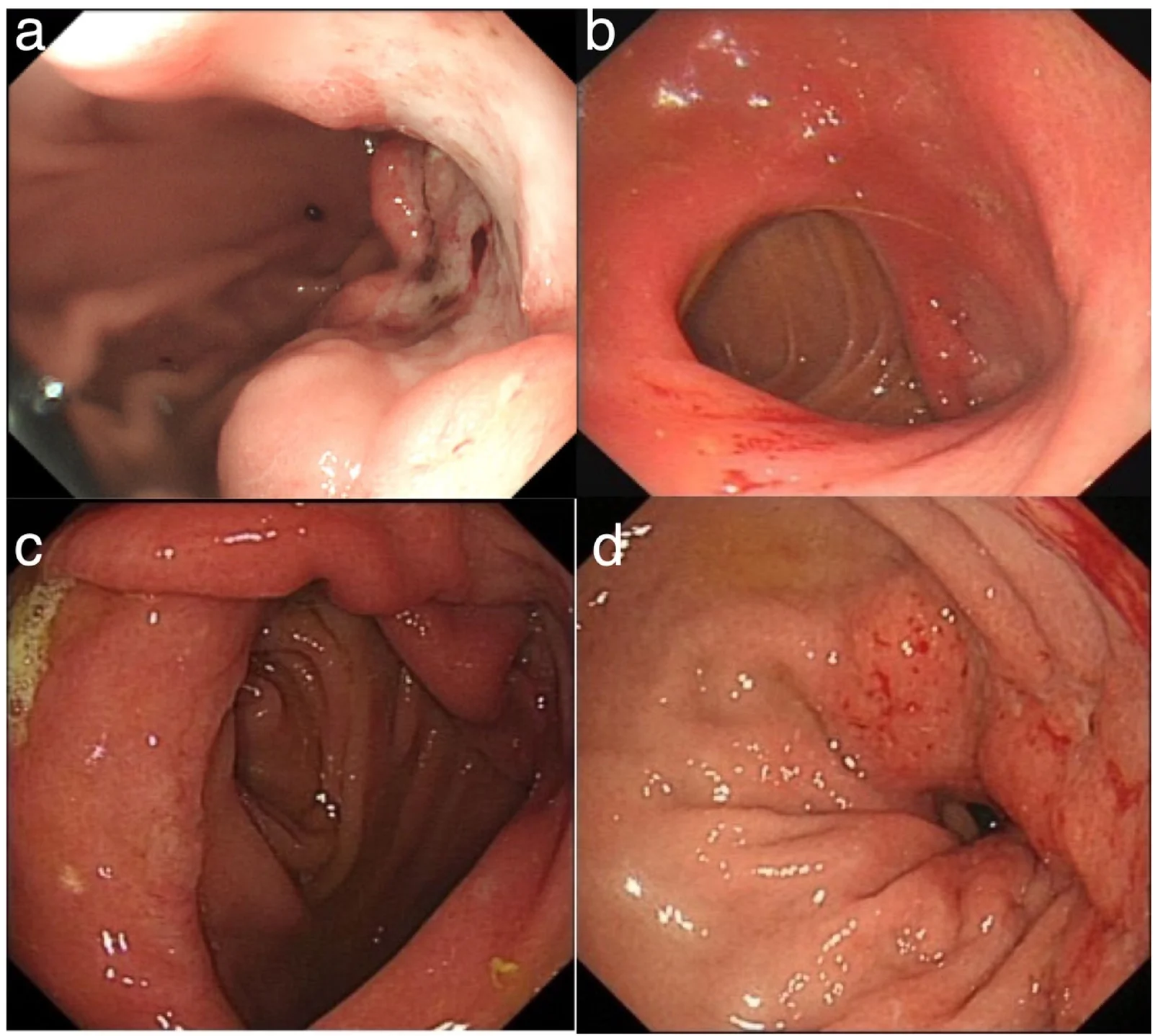
A 50-year-old woman with RGC located at the site of the anastomosis. She underwent distal gastrectomy with Billroth II reconstruction for an undifferentiated carcinoma in the lower third of the stomach (**a**). The anastomosis site was normal without obvious gastritis or bile reflux at 1-year (**b**) and 2-year (**c**) follow-up gastroscopies. Undifferentiated-type RGC emerged at the anastomotic site at 3-year follow-up gastroscopy (**d**).

**Figure 2:**
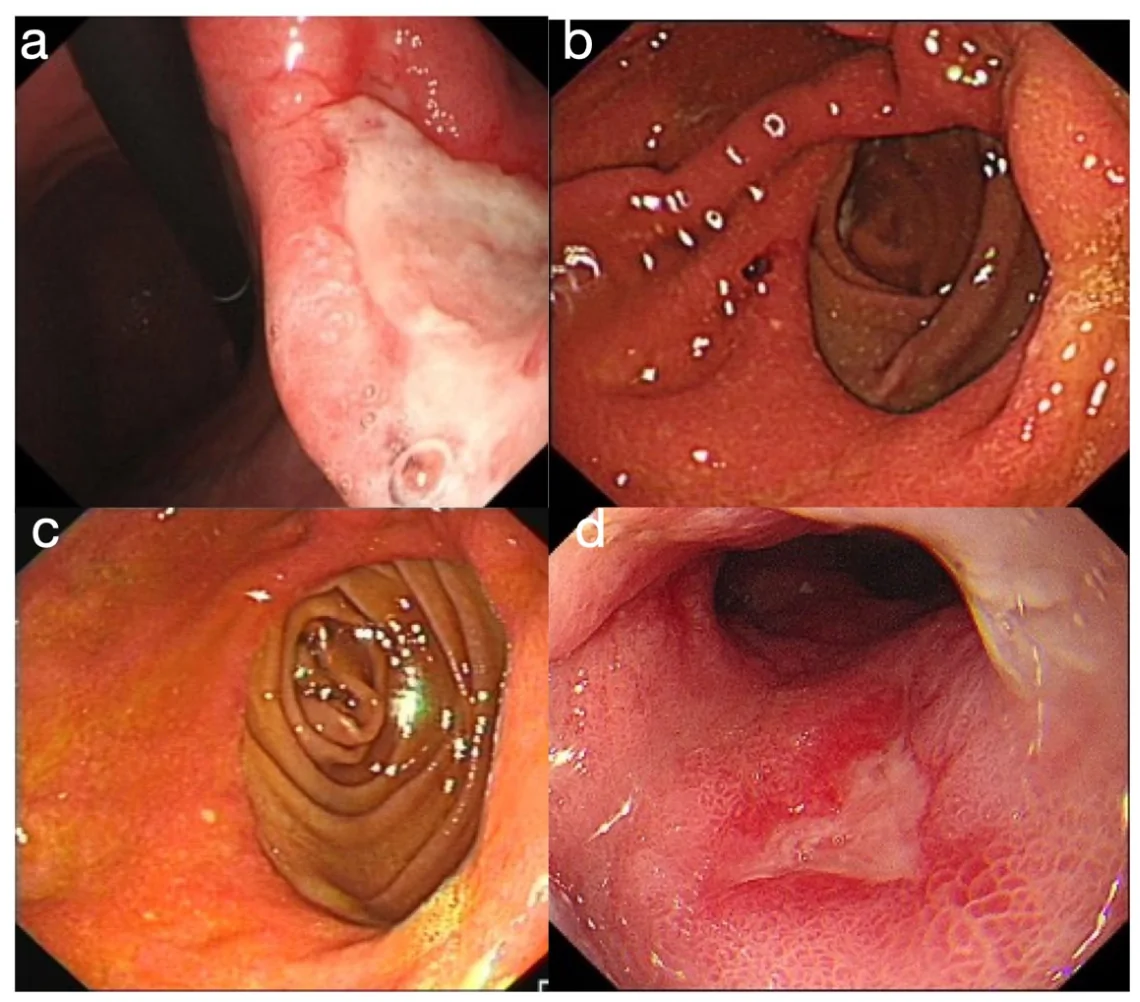
A 62-year-old man with RGC at a non-anastomotic site. The patient underwent distal gastrectomy with Billroth II reconstruction for an undifferentiated carcinoma in the lower third of the stomach (**a**). Follow-up gastroscopy at 1 year (**b**) and 2 years (**c**) showed a normal anastomosis without obvious gastritis and no visible abnormalities in the remnant gastric mucosa. At the 5-year follow-up, an undifferentiated-type RGC with surrounding gastritis was detected near the cardia at a non-anastomotic site (**d**).

The indication for the initial gastrectomy differed significantly across the three interval groups (*p* < 0.001). Gastric carcinoma was the initial disease in all 18 patients diagnosed within 3 years, in 15 of the 17 patients (88.2%) diagnosed between >3 and ≤10 years, and in only 3 of the 26 patients (11.5%) diagnosed after >10 years. Distant metastasis was documented in 11/18 (61.1%), 4/17 (23.5%), and 6/26 (23.1%) patients in the ≤3-year, >3 to ≤10-year, and >10-year groups, respectively. After patients with unknown distant-metastasis status were excluded, the overall distribution of distant metastasis differed significantly across the three interval groups (overall exact *p* = 0.022). In an exploratory unadjusted pairwise comparison restricted to patients with known distant-metastasis status, distant metastasis was more frequent in the ≤3-year group than in the >10-year group (11/17 [64.7%] vs. 6/25 [24.0%]; OR, 5.81; exact 95% CI, 1.26–27.99; *p* = 0.012). Surgery for RGC was performed in 1/18 (5.6%), 4/17 (23.5%), and 14/26 (53.8%) patients, respectively, and its frequency differed significantly across the three interval groups (overall exact *p* = 0.002). In an exploratory unadjusted pairwise comparison, surgery for RGC was less frequent in the ≤3-year group than in the >10-year group (5.6% vs. 53.8%; OR, 0.05; exact 95% CI, 0.001–0.44; *p* = 0.001). No significant differences were observed across the three interval groups in age, sex, extent of gastritis, bile reflux, tumor location, histological type, T-category distribution, or lymph-node metastasis (all *p* > 0.05; Table 4). Representative endoscopic sequences of an anastomotic RGC detected 3 years after initial gastrectomy for gastric carcinoma and a non-anastomotic RGC detected 5 years after initial gastrectomy for gastric carcinoma are shown in Figure 1 and Figure 2, respectively. Among the 18 patients diagnosed with RGC within 3 years, 13 (72.2%) had tumors at the anastomotic site, 14 (77.8%) had undifferentiated RGC, 11 (61.1%) had T4 disease, and 13 (72.2%) had lymph-node metastasis. Regarding the initial gastric carcinomas in these 18 patients, 15 (83.3%) were undifferentiated, 10 (55.6%) were classified as T4, 9 (50.0%) had lymph-node metastasis, and 3 (16.7%) had distant metastasis. Among the 18 patients diagnosed within 3 years, RGC was detected through scheduled endoscopic surveillance in nine, symptom-prompted investigation in five, and investigation following abnormalities on other examinations in two; the circumstances leading to diagnosis were insufficiently documented in two. After the initial gastrectomy, four patients (22.2%) had received postoperative chemotherapy, eight had not, and chemotherapy status was unknown in six. All had negative resection margins on final histopathological assessment. In exploratory Firth penalized logistic regression adjusted for age and sex, compared with the >10-year group, the ≤3-year group had higher odds of distant metastasis (adjusted OR, 6.50; 95% CI, 1.66–30.77; *p* = 0.007) and lower odds of surgery for RGC (adjusted OR, 0.07; 95% CI, 0.01–0.35; *p* < 0.001). The corresponding associations for the >3- to ≤10-year group were less pronounced (Supplementary Table S1). Because the indication for initial gastrectomy was strongly associated with postoperative interval, these models were not adjusted simultaneously for initial indication and therefore should not be interpreted as demonstrating an independent effect of postoperative interval.

## 4. Discussion

The principal finding of this study was the marked difference in the temporal pattern of remnant gastric cancer (RGC) according to the indication for the initial gastrectomy. Among patients whose initial gastrectomy was performed for gastric carcinoma, 50.0% of RGCs were diagnosed within 3 years, whereas 92.0% of RGCs after gastrectomy for benign disease were diagnosed more than 10 years later. The mean interval from the initial gastrectomy to RGC diagnosis was therefore substantially shorter in the carcinoma group than in the benign group. Although tumor location, histological type, T category, nodal metastasis, and distant metastasis did not differ significantly between the two indication groups, patients in the carcinoma group were less likely to undergo surgery for RGC. When patients were stratified according to postoperative interval, the ≤3-year group had a higher observed frequency of distant metastasis and a lower frequency of surgical treatment than the >10-year group. However, these groups also differed markedly in the indication for the initial gastrectomy: all patients in the ≤3-year group had initially undergone surgery for gastric carcinoma, whereas most patients in the >10-year group had undergone surgery for benign disease. Therefore, the observed differences in metastatic burden and surgical management cannot be attributed to postoperative interval alone and may also reflect differences in the biology, stage, treatment history, and recurrence potential of the original disease.

Differences in postoperative surveillance may also have contributed to the observed timing of RGC detection. Patients initially treated for gastric carcinoma were generally advised to undergo regular follow-up, whereas examinations after surgery for benign disease were predominantly prompted by symptoms or clinical indications. More frequent endoscopic surveillance could lead to earlier detection in the carcinoma group, while symptom-triggered examinations could delay detection in the benign-disease group. The observed intervals therefore reflect time to diagnosis, rather than necessarily time to tumor development. Because actual surveillance frequency and adherence could not be comprehensively reconstructed, this potential detection bias could not be adequately quantified or adjusted for. Stratification by postoperative interval does not eliminate this bias.

The remnant stomach is exposed to substantial anatomical and physiological changes after partial gastrectomy. Duodenogastric reflux, chronic mucosal inflammation, hypochlorhydria, and alterations in the local hormonal and microbial environment have been implicated in carcinogenesis within the remnant stomach, particularly after Billroth II reconstruction [3,15,16,17]. In the present cohort, 83.6% of patients had grade 2 or 3 gastritis, 72.1% had endoscopically documented bile reflux, and 63.9% had tumors at the anastomotic site. These findings should be interpreted as descriptive characteristics of patients with established RGC rather than as evidence that gastritis, bile reflux, or anastomotic location independently increases the risk of RGC. Because the study did not include a comparison group of postgastrectomy patients without RGC, neither independent associations with RGC occurrence nor causal relationships can be assessed. Similarly, although Helicobacter pylori infection and mucosal atrophy may contribute to gastric carcinogenesis, their effects could not be evaluated because *H. pylori* status and the extent of background atrophy were not systematically available. Previous evidence that *H. pylori* eradication may improve postgastrectomy mucosal inflammation and atrophic changes [18] should therefore be distinguished from direct evidence of a reduction in RGC incidence.

The long interval observed after gastrectomy for benign disease was consistent with the conventional pattern of late-onset RGC described in previous studies [3,10,11]. In our benign-disease group, 23 of 25 patients developed RGC more than 10 years after the initial operation. This pattern is compatible with a prolonged carcinogenic process within the surgically altered remnant stomach. However, because the study included only patients who eventually developed RGC and did not include the entire population undergoing Billroth II reconstruction, the observed temporal distribution cannot be used to estimate time-specific RGC incidence or determine whether the incidence of RGC increases over time. Accordingly, the concentration of cases beyond 10 years should be interpreted as a descriptive temporal pattern among patients who developed RGC rather than as evidence supporting a specific duration or interval of surveillance.

A different pattern was observed after gastrectomy for gastric carcinoma. All 18 RGCs diagnosed within 3 years occurred in patients whose initial operation was performed for carcinoma. Of these lesions, 72.2% were located at the anastomotic site and 77.8% were undifferentiated carcinomas. Furthermore, 61.1% had distant metastasis at RGC diagnosis, and only one patient underwent surgery for RGC. Gastric cancers detected within 3 years after an endoscopy that was negative for malignancy are commonly considered potentially missed lesions in endoscopy quality frameworks [19]. This definition cannot be directly applied to the present cohort, because the initial endoscopic examinations had already identified the index gastric carcinoma. Nevertheless, the five early RGCs located away from the anastomosis may have included synchronous lesions that were not recognized during the initial examination. Synchronous multiple gastric cancers have been reported in a clinically relevant proportion of patients, and small or superficial additional lesions may be overlooked when attention is focused on the dominant tumor [20,21].

The biological origin of the 13 early anastomotic lesions is more difficult to determine. Final histopathological assessment confirmed negative resection margins in all 18 patients with RGC diagnosed within 3 years. Although these findings argue against residual tumor at the assessed resection margins, they do not exclude recurrence or establish that the subsequent lesions were independent primary cancers [22,23,24]. The index tumors of patients with early-onset RGC also frequently exhibited undifferentiated histology and advanced T or nodal features, suggesting that aggressive tumor biology may have contributed to the short interval and advanced presentation. However, the present retrospective data, without a systematic review of preoperative endoscopic images, complete pathological mapping, or molecular clonality analysis, cannot determine whether these early lesions represented missed synchronous cancers, recurrent disease, or independent primary cancers. Accordingly, short-interval RGC should be interpreted as a clinically aggressive presentation rather than being assigned a single mechanism.

These temporal patterns may have implications for the design of future postgastrectomy surveillance studies but should not be interpreted as establishing a surveillance protocol. For patients undergoing partial gastrectomy for gastric carcinoma, meticulous preoperative examination of the entire stomach remains important for identifying synchronous lesions and accurately documenting tumor extent. High-quality white-light endoscopy combined with image-enhanced examination and targeted biopsies may be considered when additional lesions or indistinct tumor boundaries are suspected. The concentration of clinically advanced RGCs within the first 3 years after gastrectomy for carcinoma, together with the predominance of RGCs occurring more than 10 years after gastrectomy for benign disease, suggests that surveillance requirements may differ according to the initial indication and postoperative period. However, these observations are hypothesis-generating only. Because the present study included only patients who developed RGC, it cannot determine the optimal timing or frequency of surveillance, nor can it demonstrate that an indication- or time-based surveillance strategy improves early detection, resectability, survival, or other clinical outcomes. These questions require prospective evaluation in appropriately defined postgastrectomy cohorts.

This study has several limitations. First, the single-center retrospective design and modest sample size limited statistical power and the precision of effect estimates, particularly in subgroup analyses with sparse events. Multivariable analyses were therefore restricted to parsimonious exploratory models, and the findings should be interpreted cautiously. Although the total number of initially excluded cases was available, the numbers excluded for each individual reason could not be retrospectively reconstructed. This limited the transparency of patient selection and our ability to assess potential selection bias, which cannot be excluded. Second, because the study included only patients who developed RGC and lacked a postgastrectomy comparison cohort without RGC, neither RGC incidence nor independent risk factors could be determined. Accordingly, bile reflux, extensive gastritis, and anastomotic tumor location should be regarded as descriptive findings rather than established risk factors, and the observed temporal distribution cannot define optimal surveillance intervals or demonstrate benefit from an indication- or time-informed surveillance strategy. Third, information on the initial T category, nodal status, and distant-metastasis status was unavailable in 19 of 36 patients (52.8%) for each variable. Missing information reflected initial operations performed at other hospitals or many years previously, unavailable historical reports, records limited to brief discharge summaries, and incomplete staging documentation in the original reports. Consequently, initial tumor stage could not be adequately accounted for, and the observed associations of postoperative interval with metastatic burden and surgical treatment could partly reflect differences in the initial cancers rather than an independent effect of interval. Tumor characteristics among patients with available records may not represent the entire carcinoma group, and bias related to differential record availability cannot be excluded. The absence of standardized data on *H. pylori* status and postoperative surveillance further limited adjustment for potential confounding factors. Finally, despite verification of negative initial resection margins, absence of macroscopic residual tumor, and intervening negative endoscopic examinations, the available retrospective evidence could not reliably distinguish all short-interval new primary cancers from local recurrence or previously missed synchronous lesions. The ≤3-year subgroup may therefore include lesions of different origins, limiting interpretation of its temporal pattern and clinicopathological characteristics. Survival outcomes were unavailable; therefore, distant metastasis at RGC diagnosis should be interpreted as a measure of disease burden rather than a direct measure of prognosis. Receipt of surgery reflected actual treatment decisions, which depended on disease extent, functional status, and the anticipated benefit–risk balance, and should not be interpreted as a direct measure of oncological resectability.

In conclusion, the indication for the initial gastrectomy was strongly associated with the temporal pattern of RGC after Billroth II reconstruction. RGC after gastrectomy for gastric carcinoma frequently occurred within 3 years and was characterized by frequent distant metastasis and a lower frequency of surgical treatment, whereas RGC after gastrectomy for benign disease predominantly occurred more than 10 years after surgery. The associations with metastatic burden and surgical treatment remain exploratory because the effects of postoperative interval and initial gastrectomy indication could not be reliably separated. These hypothesis-generating findings may inform the design of future surveillance studies but do not establish an evidence-based surveillance protocol. Prospective studies including appropriate postgastrectomy comparison cohorts are required to determine optimal surveillance intervals and to evaluate whether indication- or time-informed surveillance can improve early detection or clinical outcomes.

## Figures and Tables

**Table 1:** Clinicopathological characteristics of patients with remnant gastric cancer.

Variable	Category	Overall (*n* = 61)
Age, years, mean ± SD		62.0 ± 11.0
Interval from initial gastrectomy to RGC diagnosis, years, mean ± SD		15.8 ± 16.2
Sex, *n* (%)	Male	44 (72.1%)
	Female	17 (27.9%)
Extent of gastritis	Grade 0	4 (6.6%)
	Grade 1	6 (9.8%)
	Grade 2	24 (39.3%)
	Grade 3	27 (44.3%)
Bile reflux	Present	44 (72.1%)
	Absent	17 (27.9%)
Tumor location	Anastomotic site	39 (63.9%)
	Non-anastomotic site	22 (36.1%)
Histological type	Differentiated	3 (4.9%)
	Undifferentiated	45 (73.8%)
	Unclassified	13 (21.3%)
Interval from initial gastrectomy to RGC diagnosis	≤3 years	18 (29.5%)
	>3 to ≤10 years	17 (27.9%)
	>10 years	26 (42.6%)
Surgery for RGC	Yes	19 (31.1%)
	No	42 (68.9%)

Data are presented as mean ± SD or *n* (%). RGC, remnant gastric cancer; SD, standard deviation.

**Table 2:** Comparison of clinicopathological characteristics of remnant gastric cancer according to the indication for initial gastrectomy.

Variable	Category	Initial Indication for Gastrectomy		*p* Value
		Gastric Carcinoma (*n* = 36)	Benign Disease (*n* = 25)	
Age, Years, Mean ± SD		59.0 ± 10.9	66.3 ± 9.8	0.01
Sex, *n* (%)	Male	23 (63.9%)	21 (84.0%)	0.15
	Female	13 (36.1%)	4 (16.0%)	
Extent of gastritis, *n* (%)	Grade 0	2 (5.6%)	2 (8.0%)	0.74
	Grade 1	3 (8.3%)	3 (12.0%)	
	Grade 2	13 (36.1%)	11 (44.0%)	
	Grade 3	18 (50.0%)	9 (36.0%)	
Bile reflux, *n* (%)	Present	29 (80.6%)	15 (60.0%)	0.09
	Absent	7 (19.4%)	10 (40.0%)	
Tumor location, *n* (%)	Anastomotic site	23 (63.9%)	16 (64.0%)	1.00
	Non-anastomotic site	13 (36.1%)	9 (36.0%)	
Histological type, *n* (%)	Differentiated	1 (2.8%)	2 (8.0%)	0.64
	Undifferentiated	28 (77.8%)	17 (68.0%)	
	Unclassified	7 (19.4%)	6 (24.0%)	
Interval from initial gastrectomy to RGC diagnosis, years, mean ± SD		4.8 ± 5.7	31.6 ± 12.9	<0.001
Interval from initial gastrectomy to RGC diagnosis, *n* (%)	≤3 years	18 (50.0%)	0 (0.0%)	<0.001
	>3 to ≤10 years	15 (41.7%)	2 (8.0%)	
	>10 years	3 (8.3%)	23 (92.0%)	
T category, *n* (%)	T1	2 (5.6%)	1 (4.0%)	0.66
	T2	4 (11.1%)	1 (4.0%)	
	T3	3 (8.3%)	1 (4.0%)	
	T4	22 (61.1%)	20 (80.0%)	
	TX	5 (13.9%)	2 (8.0%)	
Regional lymph node metastasis, *n* (%)	Present	23 (63.9%)	17 (68.0%)	0.45
	Absent	7 (19.4%)	2 (8.0%)	
	Unknown	6 (16.7%)	6 (24.0%)	
Distant metastasis, *n* (%)	Present	15 (41.7%)	6 (24.0%)	0.17
	Absent	18 (50.0%)	18 (72.0%)	
	Unknown	3 (8.3%)	1 (4.0%)	
Surgery for RGC, *n* (%)	Yes	7 (19.4%)	12 (48.0%)	0.03
	No	29 (80.6%)	13 (52.0%)	

Data are mean ± SD or *n* (%). Fisher’s exact test or an appropriate exact extension was used when cell counts were sparse. TX/unknown staging values were retained for descriptive reporting but excluded from inferential analyses involving the corresponding staging variable. RGC, remnant gastric cancer; SD, standard deviation.

**Table 3:** Histopathological characteristics and TNM components of the initial gastric carcinoma (*n* = 36).

Variable	Category	Patients (*n* = 36)
Histological type, *n* (%)	Differentiated	1 (2.8%)
	Undifferentiated	23 (63.9%)
	Unknown	12 (33.3%)
T category, *n* (%)	T1–T3	4 (11.1%)
	T4	13 (36.1%)
	Unknown	19 (52.8%)
Regional lymph node metastasis, *n* (%)	Present	14 (38.9%)
	Absent	3 (8.3%)
	Unknown	19 (52.8%)
Distant metastasis, *n* (%)	Present	3 (8.3%)
	Absent	14 (38.9%)
	Unknown	19 (52.8%)

Data are presented as *n* (%). Unknown indicates unavailable or insufficient information in the medical record.

**Table 4:** Comparison of clinicopathological characteristics of RGC according to the interval from initial gastrectomy.

Variable	Category	≤3 Years (*n* = 18)	>3 to ≤10 Years (*n* = 17)	>10 Years (*n* = 26)	Overall *p* Value
Age, years, mean ± SD		59.0 ± 12.2	60.1 ± 9.8	65.2 ± 10.3	0.13
Sex, *n* (%)	Male	12 (66.7%)	10 (58.8%)	22 (84.6%)	0.15
	Female	6 (33.3%)	7 (41.2%)	4 (15.4%)	
Extent of gastritis	Grade 0	0 (0%)	1 (5.9%)	3 (11.5%)	0.55
	Grade 1	1 (5.6%)	2 (11.8%)	3 (11.5%)	
	Grade 2	7 (38.9%)	5 (29.4%)	12 (46.2%)	
	Grade 3	10 (55.6%)	9 (52.9%)	8 (30.8%)	
Bile reflux	Present	14 (77.8%)	14 (82.4%)	16 (61.5%)	0.32
	Absent	4 (22.2%)	3 (17.6%)	10 (38.5%)	
Tumor location	Anastomotic site	13 (72.2%)	10 (58.8%)	16 (61.5%)	0.71
	Non-anastomotic site	5 (27.8%)	7 (41.2%)	10 (38.5%)	
Histological type	Differentiated	1 (5.6%)	0 (0%)	2 (7.7%)	0.82
	Undifferentiated	14 (77.8%)	12 (70.6%)	19 (73.1%)	
	Unclassified	3 (16.7%)	5 (29.4%)	5 (19.2%)	
Indication for initial gastrectomy	Gastric carcinoma	18 (100%)	15 (88.2%)	3 (11.5%)	<0.001
	Benign disease	0 (0%)	2 (11.8%)	23 (88.5%)	
Depth of tumor invasion (T)	T1	1 (5.6%)	1 (5.9%)	1 (3.8%)	0.61
	T2	1 (5.6%)	2 (11.8%)	2 (7.7%)	
	T3	3 (16.7%)	0 (0.0%)	1 (3.8%)	
	T4	11 (61.1%)	11 (64.7%)	20 (76.9%)	
	TX	2 (11.1%)	3 (17.6%)	2 (7.7%)	
Lymph-node metastasis	Positive	13 (72.2%)	10 (58.8%)	17 (65.4%)	0.90
	Negative	3 (16.7%)	3 (17.6%)	3 (11.5%)	
	Unknown	2 (11.1%)	4 (23.5%)	6 (23.1%)	
Distant metastasis	Positive	11 (61.1%)	4 (23.5%)	6 (23.1%)	0.022
	Negative	6 (33.3%)	11 (64.7%)	19 (73.1%)	
	Unknown	1 (5.6%)	2 (11.8%)	1 (3.8%)	
Surgery for RGC	Yes	1 (5.6%)	4 (23.5%)	14 (53.8%)	0.002
	No	17 (94.4%)	13 (76.5%)	12 (46.2%)	

Data are presented as mean ± SD or *n* (%). Overall *p* values represent comparisons among the three interval groups. Categorical variables were analyzed by χ^2^ or exact tests, as appropriate; TX/unknown staging values were retained for descriptive reporting but excluded from inferential analyses involving the corresponding staging variable. In exploratory ≤3- vs. >10-year comparisons, unadjusted ORs (exact 95% CIs) were 5.81 (1.26–27.99) for distant metastasis (*p* = 0.012) and 0.05 (0.001–0.44) for surgery for RGC (*p* = 0.001). Pairwise tests were unadjusted for multiplicity and should be interpreted as exploratory; adjusted analyses are shown in Supplementary Table S1. RGC, remnant gastric cancer; SD, standard deviation.

## Data Availability

The data are not publicly available due to patient privacy and ethical restrictions.

## Supplementary Materials

The following supporting information can be downloaded at: https://www.mdpi.com/article/10.3390/jcm15207933/s1, Table S1: Exploratory adjusted associations of postoperative interval with distant metastasis and surgery for remnant gastric cancer.

## Abbreviations

AJCC: American Joint Committee on Cancer; RGC: remnant gastric cancer; SD: standard deviation; TNM: tumor-node-metastasis; WHO: World Health Organization.
