# Supplementary material for: Initial Gastrectomy Indication Is Associated with Divergent Temporal Patterns of Remnant Gastric Cancer After Billroth II Reconstruction: A Retrospective Study
Source: J Clin Med. 2026 Oct 14;15(20):7933. doi: 10.3390/jcm15207933 (PMC13705669; doi:10.3390/jcm15207933)
Supplement: Supplementary file 1 [file jcm-15-07933-s001.zip › jcm-4552142-supplementary.pdf]

Supplementary Table S1. Exploratory adjusted associations of postoperative interval with distant metastasis and surgery for remnant gastric cancer

Outcome	Postoperative interval	Events/total, n/N	Adjusted OR (95% CI)	P value
Distant metastasis at RGC diagnosis (n = 57)	≤3 years	11/17	6.50 (1.66–30.77)	0.007
	>3 to ≤10 years	4/15	1.44 (0.32–6.28)	0.629
	>10 years	6/25	Reference	—
Surgery for RGC (n = 61)	≤3 years	1/18	0.07 (0.01–0.35)	<0.001
	>3 to ≤10 years	4/17	0.25 (0.06–0.94)	0.040
	>10 years	14/26	Reference	—

Note: Data are adjusted odds ratios (ORs) with 95% confidence intervals (CIs). Firth penalized logistic regression was used because of the modest sample size and sparse events. Models were adjusted for age and sex, with the >10-year group as the reference category. Patients with unknown distant-metastasis status were excluded from the distant-metastasis model (n = 57); all 61 patients were included in the surgery model. RGC, remnant gastric cancer.
